# Enhanced Antitumor and Antibacterial Activities of Ursolic Acid through β-Cyclodextrin Inclusion Complexation

**DOI:** 10.1021/acsomega.4c08337

**Published:** 2025-03-26

**Authors:** Júlia B. Fajardo, Mariana H. Vianna, Thayná G. Ferreira, Ari S. de O.Lemos, Thalita de F. Souza, Lara M. Campos, Priscila de L. Paula, Nubia B. Andrade, Lívia R. Gamarano, Lucas S. Queiroz, Bruno de A. Oliveira, Adilson D. da Silva, Luciana M. Chedier, Ângelo M. L. Denadai, Guilherme D. Tavares, Thaís N. Barradas, Rodrigo L. Fabri

**Affiliations:** † Laboratory of Bioactive Natural Products, Department of Biochemistry, Institute of Biological Sciences, Federal University of Juiz de Fora, Juiz de Fora, Minas Gerais CEP 36036-900, Brazil; ‡ Research Group for Food Production Engineering, National Food Institute, Technical University of Denmark, Ørsteds Plads, Kongens Lyngby 2800, Denmark; § Department of Chemistry, Institute of Exact Sciences, Federal University of Juiz de Fora, Juiz de Fora, Minas Gerais CEP 36036-900, Brazil; ∥ Department of Botany, Institute of Biological Sciences, Federal University of Juiz de Fora, Juiz de Fora, Minas Gerais CEP 36036-900, Brazil; ⊥ Department of Pharmacy, Institute of Life Sciences, Federal University of Juiz de Fora, Campus Governador Valadares, Juiz de Fora, Minas Gerais CEP 36036-900, Brazil; # Department of Pharmacy, Faculty of Pharmacy, Federal University of Juiz de Fora, Juiz de Fora, Minas Gerais CEP 36036-900, Brazil

## Abstract

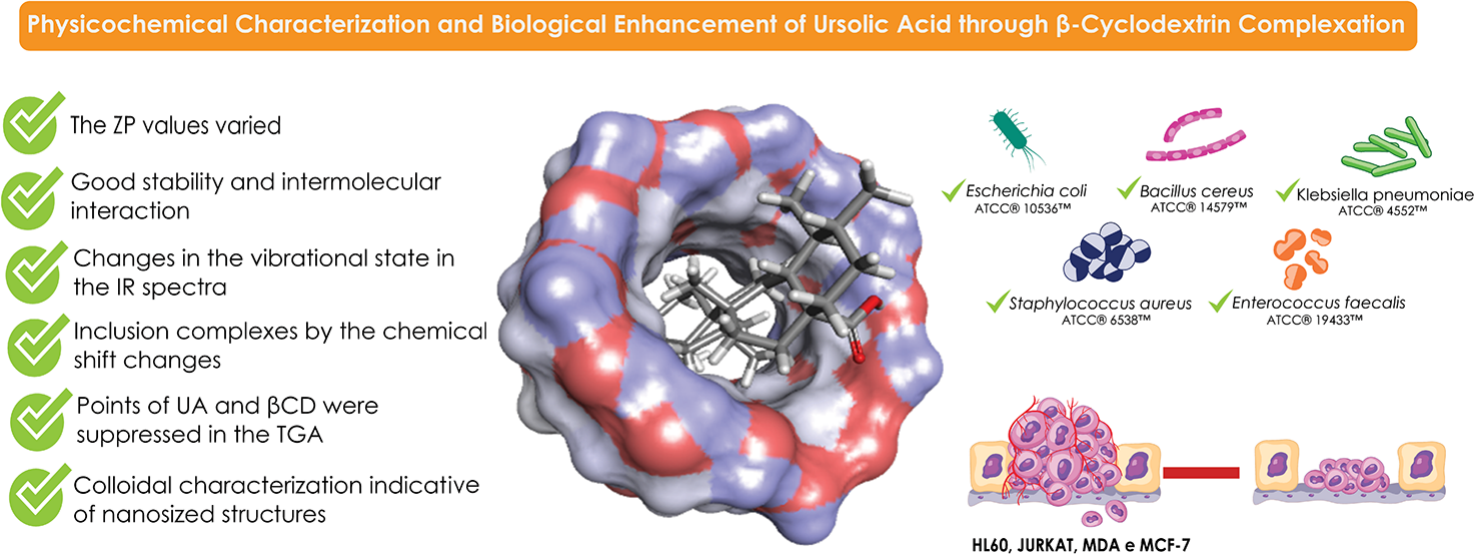

Ursolic acid (UA) is a pentacyclic triterpenoid known for its wide range of biological activities, including anticancer and antimicrobial effects. However, its poor solubility in water limits its therapeutic potential. Therefore, this work aims to evaluate the physicochemical properties of the ursolic acid/β-cyclodextrin inclusion complex (UA/βCD IC) and investigate the enhancement of the *in vitro* antitumor and antibacterial activities of UA when complexed with βCD. Molecular docking simulation showed that the carbonyl group of UA binds to the internal cavity of βCD, forming a hydrogen bond with the glucosidic residues of βCD. FTIR analysis revealed significant changes in the absorption peaks of UA/βCD IC, indicating interaction between the compounds, such as the reduction in intensity of the C=O and ν(O–H) bands. These results were supported by thermal analysis, as the degradation temperature of UA (233°C) and βCD (294°C) was suppressed in UA/βCD IC (191°C) compared to the free components. In addition, NMR analysis revealed significant changes in the chemical shift of the H located on the anomeric carbon (C1) of the glucose units in β-CD for the IC spectra (Δδ: 0.0041 ppm) compared to βCD, which are related to perturbations in the atomic electronic density. The colloidal characterization results also showed that UA/βCD IC has more stable colloidal properties with higher zeta potential values compared to free UA. As shown by the solubility assay, the interaction between UA and βCD formed stable inclusion complexes that increased the aqueous solubility of UA by approximately 35.85% (AUC: UA = 12.72, βCD = 6.78, UA/βCD = 17.28, *p* < 0.05). Scanning electron microscopy images revealed that IC was also associated with significant changes in particle shape and size. In addition, the UA/βCD IC showed greater antitumor activity than free UA, particularly in the MDA (71.95 ± 4.88%) and MCF-7 (73.40 ± 1.55%) cell lines. It showed similar efficacy to etoposide in HL60 (86.9 ± 0.84%) and JURKAT (85.35 ± 4.03%) cells. The UA/βCD IC significantly reduced the MIC values, improving the antibacterial activity particularly against *E. faecalis* (UA MIC: 31.3 μg/mL; UA/βCD MIC: 7.8 μg/mL), followed by *S. aureus*, *B. cereus*, and *K. pneumoniae* (UA MIC: 31.3 μg/mL; UA/βCD MIC: 15.6 μg/mL). Therefore, the UA/βCD IC significantly modifies the physicochemical properties of UA, resulting in enhanced aqueous solubility and biological properties, as confirmed by the improved antitumor and antibacterial activities.

## 1 Introduction

Ursolic acid (UA) is a pentacyclic triterpenoid with a molecular weight of 456.70 g/mol. It is found in various plant species and may occur as free acids or serve as aglycones for triterpene saponins.^1^ Over the past few years, UA has demonstrated interesting biological activities, including anticancer, anti-inflammatory, antiviral, antimicrobial, antidiabetic, antihypertensive, antihyperlipidemic, analgesic, hepatoprotective, gastroprotective, antiulcer, anti-HIV, antiatherosclerotic, and immunomodulatory effects.^2^

Studies suggest that UA inhibits cell proliferation and metastasis of cancer cells through various pathways, such as the PI3K/Akt/mTOR pathway in ovarian cancer, the JAK/STAT pathway in prostate cancer, the NF-κB pathway in gastric cancer, and the MAPK pathway in melanoma and HeLa cells.^3^ According to Kang et al., 2021,^4^ UA can also inhibit the proliferation of A549 and H460 lung cancer cells by disrupting the G0/G1 cell cycle, leading to apoptosis. In addition, several reports have demonstrated the antimicrobial activity of UA. Qian et al., 2020^5^ showed that UA was effective against carbapenem-resistant *Klebsiella pneumoniae* by disrupting the cell membrane and inhibiting biofilm formation. Its antimicrobial and antibiofilm activities have also been observed against *Staphylococcus aureus*, *Acinetobacter baumannii*, *Escherichia coli*, and *Streptococcus mutans*.^6^

However, the poor solubility of UA in aqueous solutions limits its bioavailability and practical application in pharmaceutical formulations. In addition, UA can be quickly metabolized in the liver and exhibits nonspecific distribution in the body, which impairs UA plasma half-life and enhances adverse effects caused by it.^7^ In this regard, innovative approaches are needed to enhance UA selectivity and hydrophilicity, improving UA bioavailability, therapeutic efficacy, and clinical application. Previous studies have shown that the aqueous solubility and bioavailability of UA can be significantly improved by encapsulation in dendrimer nanoparticles and by incorporation of the compound into an acid-phospholipid complex. Other strategies may also be considered, such as the development of liposomes, inclusion complexes, and chemical modifications.^8,9^ UA’s chemical structure includes a hydrophobic triterpene backbone, which contributes to its low aqueous solubility, making complexation with cyclodextrins a suitable strategy for improving its solubility and stability.

Cyclodextrins (CDs) are cyclic oligosaccharides widely used in the pharmaceutical and food industries due to their interaction profile with poorly soluble molecules. Such interactions lead to the formation of supramolecular inclusion complexes, which can improve physicochemical properties such as stability, solubility, dissolution rate, and bioavailability of the entrapped molecules. Furthermore, supramolecular inclusion complexes (ICs) can promote controlled drug release, reducing drug toxicity and the occurrence of adverse effects.^10−12^

Previous studies using physicochemical characterization have shown that UA spontaneously interacts with the CD cavity. These interactions result in a stable IC that can increase aqueous solubility and UA stability. Furthermore, they have also demonstrated that complexing biological compounds with βCD can enhance their anti-proliferative activity compared to the free molecules.^13−15^ This work aimed to evaluate the physicochemical properties of the UA/βCD inclusion complex and the improvement of *in vitro* antitumor and antibacterial activities of UA in the presence of βCD.

## 2 Materials and Methods

### 2.1 Isolation of Ursolic Acid (UA) from *Mitracarpus frigidus* Aerial Parts

UA, used in this study, was previously isolated from the aerial parts of *Mitracarpus frigidus* (Willd. ex Roem. & Schult.) K. Schum. Its structural elucidation was performed by EI mass spectra, IR, ^1^H-NMR, COSY, HMBC, HSQC, ^13^CNMR, and DEPT 135.,^16^ with a confirmed purity of ≥ 95%, as verified by high-performance liquid chromatography (HPLC-DAD).

### 2.2 Molecular Docking Simulations

In the first step, we used the ChemSketch program v11.0 (ACD, 2015) to draw the UA (2D structure), and its 3D structure was minimized using the MMFF94s force field in the Avogadro program v1.1.1. In the next step, the UA was prepared using the AutoDockTools program v1.5.6, and its PDBQT file was generated. βCD was used as the rigid receptor, and the guest molecule (UA) was used as the rigid ligand. The three-dimensional structure of βCD was extracted^17^ from the RCSB Protein Data Bank PDB file 3CGT. The structure of βCD was prepared using the AutoDockTools program v1.5.6 as well, where hydrogens were added, and the Gasteiger charges were computed. Molecular docking was performed with the AutoDock Vina program v1.1.2. The size of the grid was X = Y = Z = 20 Å (center: X = 0.065 Å, Y = 77.233 Å, Z = 173.031 Å), using a discretization of 0.375 Å. The number of conformations observed in the simulation was 20. We classified the docking results according to their score values and the interactions formed between βCD and UA using the PyMOL program v1.8.2.1. The images were generated using the PyMOL program v1.8.2.1 as well.

### 2.3 Preparation of the Inclusion Complexes

The inclusion complex (IC) resulting from the complexation of UA with βCD (Sigma-Aldrich, C_42_H_70_O_35_, MMw = 1134.98, purity >97%) was prepared by the co-precipitation method at a molar ratio of 1:1.^18−20^ Briefly, equimolar amounts of UA and βCD (1:1) were dissolved in a 50:50 ethanol/water mixture, which was stirred for 24 h and subsequently concentrated in a rotary evaporator at approximately 50 °C. The sample was then frozen and lyophilized for later use. Nominal concentrations of UA and βCD (equivalent weight ratio of UA/βCD 1:1) were used for the chemical and biological characterization. The selection of the 1:1 molar ratio for the UA and βCD inclusion complex was based on previous solubility tests conducted prior to the main study. These preliminary solubility assessments indicated that the 1:1 ratio provided optimal results in terms of enhancing the solubility of UA and has also been commonly reported in the literature for similar complexes.^21,22^

### 2.4 Spectroscopic Characterization of the Inclusion Complex

FTIR spectra were obtained in transmission mode by using a PerkinElmer Spectrum Two FTIR spectrometer. The components, i.e., UA, βCD, UA/βCD, and physical mixture (PM), were prepared in KBr pellets, and measurements were carried out between 4000 and 400 cm^–1^ with 16 scans and a resolution of 2 cm^–1^. For the treatment of the spectra, PerkinElmer Spectrum ES software (version 10.03.08.0133) was used. The final figures were transferred to Microcal Origin 8.0.

### 2.5 Differential Thermal Analysis (DTA) and Thermogravimetric Analysis (TGA)

Differential thermal analysis (DTA) and thermogravimetric analysis (TGA) were performed using the TGA/DTA module STA7200RV from HITACHI, coupled with a photo visualization system. Data were recorded for UA, βCD, UA/βCD, and PM at a weight ratio of 1:1. The experiments were conducted under a dynamic air atmosphere of 50 mL/min, with a heating rate of 10 °C/min and a sensitivity of 1.0 °C. For each experiment, approximately 2 mg of the sample was used and placed in open platinum pans.^23,24^

### 2.6 Determination of Particle Size by Dynamic Light Scattering (DLS)

Stock solutions were prepared by the initial dissolution of 20 mg/mL UA and 40 mg/mL UA/βCD in dimethyl sulfoxide (DMSO). Subsequently, 42 injections of 5 μL of these solutions were titrated into a larger volume of ultrapure water (1.5 mL), and hydrophobic nanoprecipitates (HNPs) were spontaneously formed by a simple mixture.

A Malvern Zetasizer Nano ZS particle analyzer using polyethylene square cells was used to measure the average hydrodynamic diameter (*D*_h_) of UA and UA/βCD via dynamic light scattering (DLS) experiments. The solutions were subjected to monochromatic light (4 mW He–Ne laser, wavelength 633 nm), and the scattered light intensity was measured at an angle of 90°. Each point was the average of 5 independent measurements, with each measurement being the mean of 30 counts.^24^

### 2.7 Zeta Potential (ZP) Measurement

Zeta potential (ZP) experiments were also performed by titration under conditions similar to those of the DLS experiments. Malvern Zetasizer Nano ZS90 equipment was used for these experiments. The ZP was determined using the laser Doppler microelectrophoresis technique at a scattering angle of 173° with a disposable folded capillary cell (DPS1060). The ZP values were calculated as the average of 10 independent measurements, with each measurement being the mean of 10 counts.

### 2.8 Scanning Electron Microscope (SEM)

βCD, UA, UA/βCD, and PM were dried for 24 h. These samples were fixed on the copper sample table with adhesive tape and then placed into the vacuum plating apparatus cover to spray gold with an acceleration voltage of 20 kV and a magnification of 10 K, followed by automatic filming using the workstation . The main technical specifications of the instrument (JEOL JSM-6390LV, Tokyo, Japan) are as follows: a resolution of 0.6 nm, a magnification range of 20–200000, and a voltage range of 1–30 kV.

### 2.9 Nuclear Magnetic Resonance Spectroscopy

Nuclear magnetic resonance (NMR) spectra were acquired at 25 °C by using a Bruker AVANCE III 500 MHz spectrometer. Chemical shifts (δ) were expressed as ppm relative to tetramethylsilane. βCD, UA/βCD IC, and PM were dissolved in DMSO-*d*_*6*_ (Sigma-Aldrich) at a final concentration of 10 mg/mL. In the spectra, two strong signals observed at δ = 2.50 and δ = 3.36 ppm were attributed, respectively, to the isotopic form of DMSO and DMSO-*d*_*6*_ residual H_2_O.

### 2.10 Solubility Evaluation

Solubility evaluation was performed according to the method reported by Higuchi and Connors (1965) with modifications.^25^ Aqueous saturated solutions of UA/βCD (1:1 molar ratio), UA, and βCD were prepared and stirred for 24 h at room temperature in the dark. The samples were then filtered through a 0.45 μm PTFE membrane filter and analyzed using a UV-vis spectrophotometer (Thermo Scientific SkanIt Multiskan GO, software version 3.2) in the wavelength range of 200–400 nm. After filtration, the terpenoid content was measured according to the method reported by Pedrosa et al.^26^ with modifications. Briefly, 1 mL of each solution was dried and resuspended in vanillin and sulfuric acid. The samples were kept in a water bath at 60 °C for 30 min and then cooled in an ice bath for 20 min. Absorbance was measured at 548 nm. The experiment was performed in triplicate.

### 2.11 Evaluation of Cytotoxic Effects against Human Cell Lines

Cancer cell (HL60, JURKAT, MDA, and MCF-7) and normal cell (VERO) lines were grown in culture bottles with RPMI-1640 medium supplemented with 2 mM l-glutamine, 100 μg/mL antibiotics (streptomycin and penicillin), and 5% fetal bovine serum (FBS) and kept in an incubator at a 5% CO_2_ atmosphere at 37 °C until the day of the test. After this period, the cells were transferred to 96-well microplates and treated with βCD, UA, and UA/βCD at 30 μg/mL for 24 h. UA, βCD, and UA/βCD were previously dissolved in dimethyl sulfoxide (DMSO) and then diluted in aqueous media to a final concentration of 0.06% DMSO. For the positive control, cells were treated with etoposide (ETO). The cells were incubated for 48 h at 37 °C under a 5% CO_2_ atmosphere. Cytotoxicity was assessed by cell viability using the 3-(4,5-dimethyl-2-thiazolyl)-2,5-diphenyl-2*H*-tetrazolium bromide (MTT) assay.^27^ Absorbance was measured at 570 nm. The experiments were performed in triplicate.

### 2.12 Evaluation of Antibacterial Activity

The antibacterial activity of UA, βCD, and UA/βCD was evaluated according to CLSI guidelines to determine the minimal inhibitory concentration (MIC).^28^ Strains of *Staphylococcus aureus* ATCC 6538, *Bacillus cereus* ATCC 14579, *Klebsiella pneumoniae* ATCC 4552, *Enterococcus faecalis* ATCC 19433, and *Escherichia coli* ATCC 10536 were grown at 35 °C for 24 h on Mueller Hinton agar. The stock solutions of UA, βCD, and UA/βCD were diluted in 1% DMSO with concentrations ranging from 1000 to 3.9 μg/mL. Adjusted bacterial concentrations (10^6^ CFU/mL, 0.5 McFarland’s standard) were used to determine MIC in Mueller Hinton broth. Ciprofloxacin and chloramphenicol (12.5 to 0.097 μg/mL) were used as positive controls. The plates were incubated at 35 °C for 24 h. The MIC endpoint represents the lowest concentration of the sample at which no visible growth is observed To determine the minimum bactericidal concentration (MBC), a sample from each well that showed no visible bacterial growth in the MIC assay was plated on freshly prepared Mueller Hinton agar plates and subsequently incubated at 35 °C for 24 h. The MBC was expressed as the concentration of the sample that did not show any growth on a new set of agar plates. The experiments were performed in triplicate.

### 2.13 Statistical Analysis

Statistical analysis was performed by one-way ANOVA followed by the Bonferroni test using the software GraphPad Prism 5.0. Values of *p* < 0.05 were considered significant. Results were expressed as the mean ± standard deviation.

## 3 Results and Discussion

### 3.1 Molecular Docking

**Figure 1:**
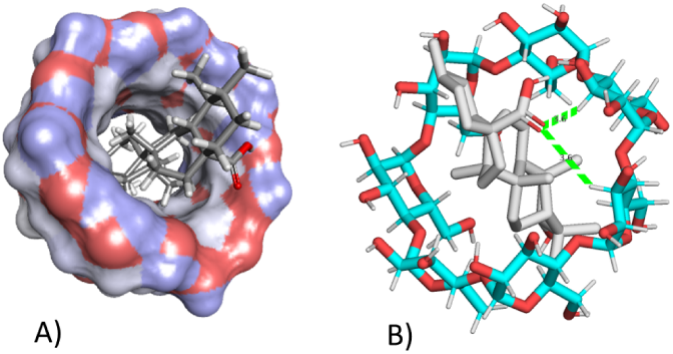
Evaluation of the interaction of ursolic acid with βCD by molecular docking. A) Interaction of ursolic acid with the inner cavity of βCD. B) Hydrogen bonding of the carbonyl group of ursolic acid with the hydrogens of glucose residues present in βCD (dashed green lines).

Using the molecular docking technique, it was observed that UA showed a binding interaction (−6.5 kcal/mol) with the internal cavity of the βCD (Figure 1A). Furthermore, it is possible that this interaction is a consequence of the presence of a carbonyl group forming a hydrogen bond (3.6 Å) with the glucosyl residues of the βCD (Figure 1B). These results show that UA has achieved total inclusion with βCD through hydrogen bonding and hydrophobic interactions such as van der Waals forces. These bonds and interactions occur because ursolic acid has a carbonyl group that has the ability to bind to the βCD cavity (the O atom in the carbonyl group of UA bonded to the H atoms attached to the C3 atom in glucosyl residues G4 and G5, respectively, inside the cavity of βCD) through intermolecular hydrogen bonding and van der Waals interactions.^29^

### 3.2 FTIR Experiment

**Figure 2:**
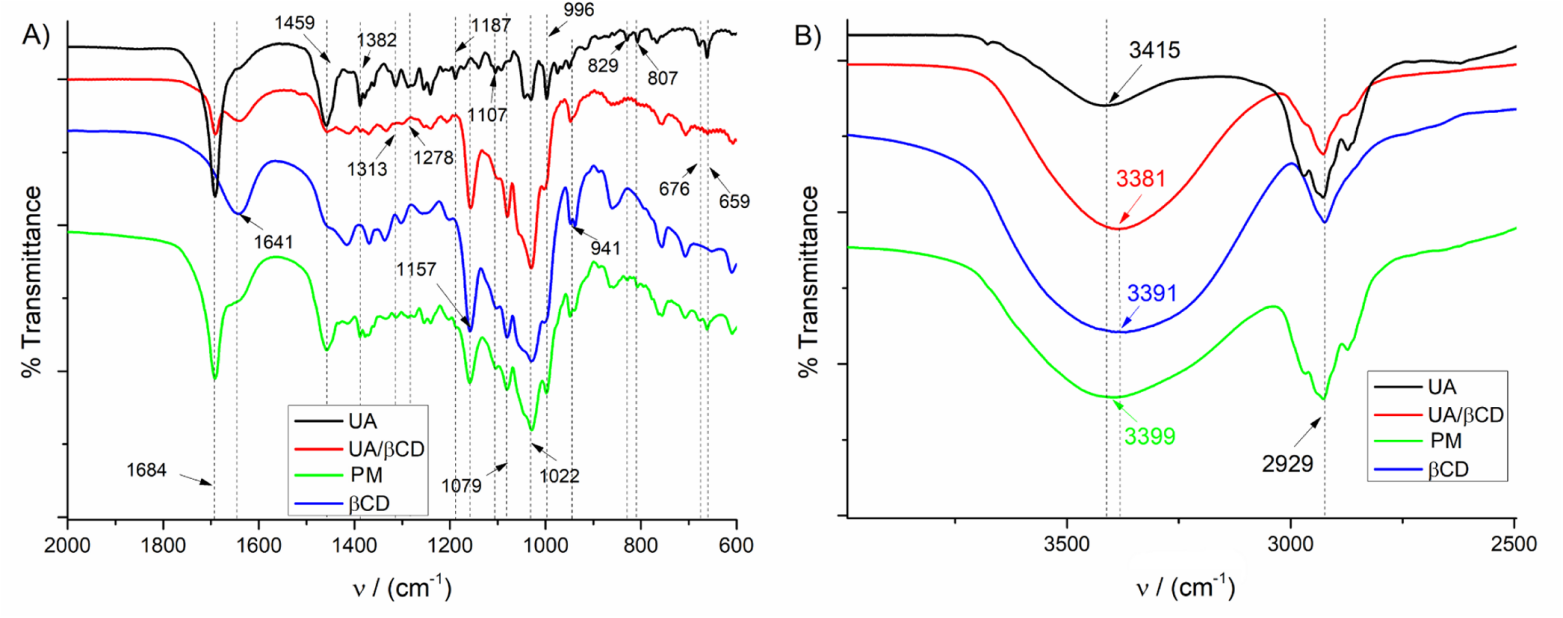
FTIR spectra of UA, UA/βCD, PM, and βCD in transmittance mode. A) Range from 600 to 200 cm^–1^ and B) range from 2500 to 4500 cm^–1^. UA = ursolic acid; UA/βCD = UA/βCD inclusion complex; PM = physical mixture between βCD and UA; βCD = β-cyclodextrin.

The FTIR spectra of UA, βCD, UA/βCD, and PM (Figure 2A,B) were used to confirm the formation of the inclusion complex in the solid state. The bands of the βCD spectrum agree with those already described in the literature, with main absorptions at 3391, 2929, 1641, 1187, 1157, 1079, 1022, 941, 829, and 807 cm^–1^, which correspond to the symmetric and antisymmetric stretching of ν[OH], ν[CH2], ν[C–C], the bending vibration of ν[O–H], and the skeletal vibration involving (α-1,4 linkage), respectively.^30^

The primary alcohol groups of βCD can be observed in the range of 1056–1028 cm^–1^, which is related to the characteristic bond C–O from the βCD chemical structure. The spectrum of βCD also revealed distinctive features, such as a noticeable broad band observed at around 3700 and 3100 cm^–1^, which can be attributed to the stretching vibrations of ν(O–H) [23]. The UA spectrum has shown main absorption bands corresponding to the symmetric and antisymmetric stretching of ν[O–H], ν[CH2], ν[C=O], ν[O–H], ν[CH3], and ν[C–O] at around 3415, 2932, 1684, 1459, 1382, 1107, and 996 cm^–1^, respectively.^30−32^

In the UA/βCD spectrum, it is possible to detect significant changes in the shape and intensity of FTIR peaks as a result of the inclusion of molecules, which have been attributed to the vibrational restriction of molecules upon inclusion. The reduction in the intensity of the C=O related band, at around 1684 cm^–1^, confirms the interaction of UA and βCD, corroborating with the molecular docking results. Moreover, a reduction in the intensity of bands at around 1460 cm^–^,^1^ which correspond to the vibrational mode of ν(O–H), was noted, indicating interaction between UA and βCD.^33^ The FTIR spectra revealed a distinct change in the UA carbonyl peak when comparing the pure drug to the PM and the IC, in which the band corresponding to the carbonyl peak appears reduced and broadened, in contrast to the sharp peak observed in the pure drug or PM. In addition, a displacement of hydroxyl bands (stretching of ν[OH]) close to 3400 cm^–1^) is observed for the inclusion compound, compared with pure βCD and UA. This alteration was attributed to the formation of a new pattern of hydrogen bonding after interactions between the compounds in the solid state.^34^

### 3.3 Thermogravimetric Analysis and Differential Thermal Analysis

**Figure 3:**
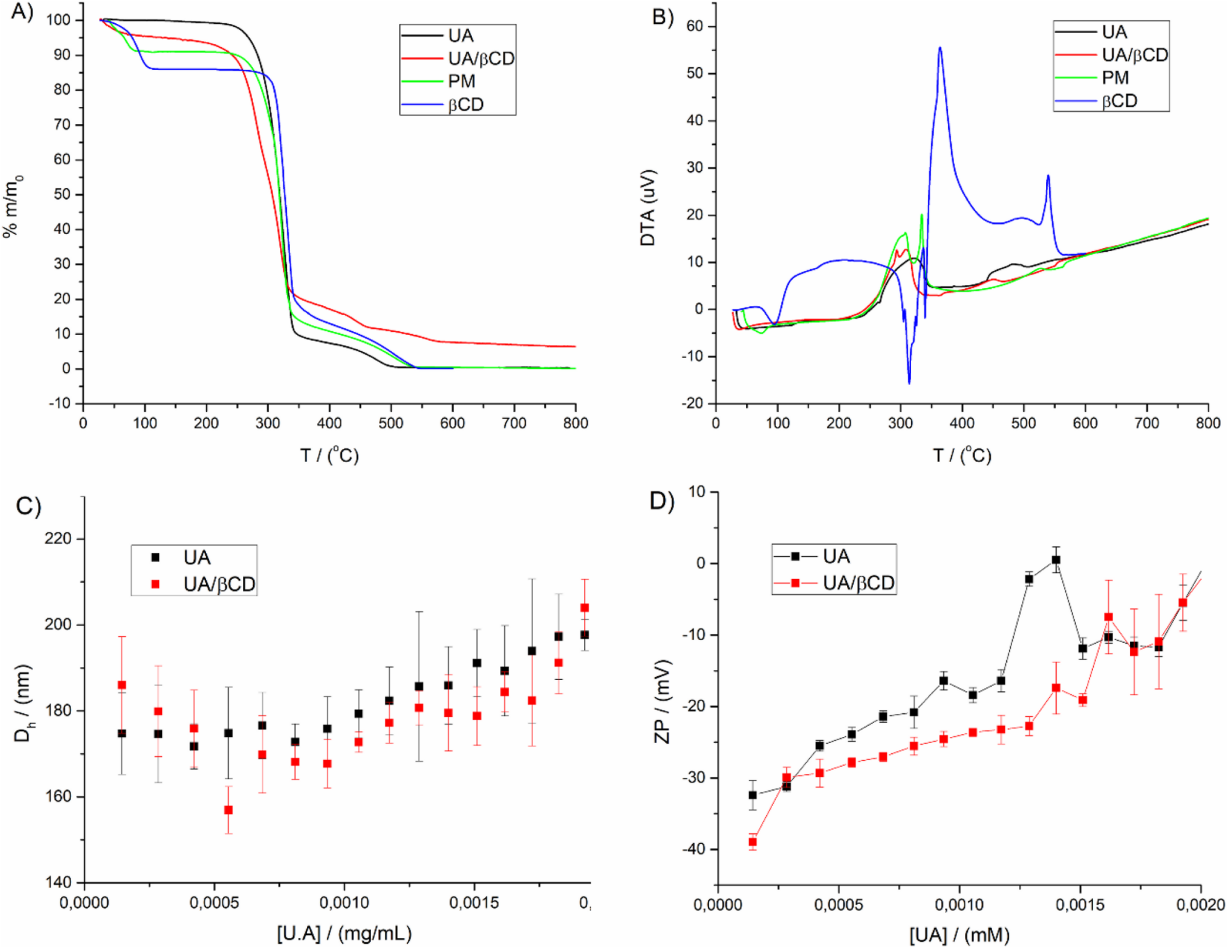
A) TGA curve of UA, UA/βCD, PM, and βCD in temperatures ranging from 30 to 800 °C. B) DTA curve of UA, UA/βCD, PM, and βCD in temperatures ranging from 30 to 800 °C. C) Distribution of the *D*_h_ values measured for different UA and UA/βCD concentrations. D) Distribution of the ZP values measured for different UA and UA/βCD concentrations. UA = ursolic acid; UA/βCD = UA/βCD inclusion complex; PM = physical mixture between βCD and UA; βCD = β-cyclodextrin.

Figures 3A,B shows, respectively, the thermogravimetric analysis (TGA) and differential thermal analysis (DTA) curves for UA, UA/βCD, PM, and βCD. The TGA curve for βCD shows a mass loss of 13.8% in the range of 64 to 115 °C, corresponding to the release of approximately 12 water molecules. The DTA curve shows an endothermic peak within this range, confirming the dehydration phenomenon. Following this event, thermal stability was observed in both TGA and DTA up to approximately 290 °C, at which point the decomposition of βCD begins.^35^

The DTA curve for UA shows a melting transition at about 265 °C, resulting from a decomposition phenomenon, as the TGA curve for UA shows a sharp loss in the same temperature range. Immediately after this first decomposition temperature, the UA suffers another loss of mass until its complete calcination at ≈ 500 °C.

The physical mixture showed three mass loss steps: (i) the first one, between 41 and 88 °C, was due to the dehydration phenomenon from the βCD cavity, with 10.3% of weight loss; (ii) the second one, between 229 and 360 °C, with 77.7% of weight loss, was due to UA decomposition; and (iii) the last, between 360 and 554 °C, with 5.6% of weight loss, was due to both UA and βCD degradation. Such an observation, together with our previous results from FTIR, reflects the heterogeneous material derived from PM.

For the IC, the thermal profile is different from that observed for pure precursors and PM as a result of the complexation. In the TGA curve, it can be observed that the UA/βCD sample lost approximately 5.2% of its mass up to 84 °C, with this phenomenon attributed to the release of remaining water molecules after inclusion. Moreover, as can be seen in the DTA curve, the UA and the βCD had their melting transitions suppressed as a result of intermolecular interactions.

Additionally, the degradation temperature for the UA/βCD IC (191 °C) is significantly lower than that of the UA (233 °C) and PM (229 °C), corroborating the existence of interactions in the solid state. The detailed values of onset, endset, and maximum temperatures for each weight loss step are provided in Table 1.

**Table 1:** Thermogravimetric Data for UA, βCD, PM, and UA/βCD from ≈30 to 800 °C in Air Atmosphere^a^

	Dehydration				1^st^ Decomposition				2^rd^ Decomposition				
Sample	%Δ*m*	*T*_onset_	*T*_endset_	*T*_max_	%Δ*m*	*T*_onset_	*T*_endset_	*T*_max_	%Δ*m*	*T*_onset_	*T*_endset_	*T*_max_	Δ*m*_total_
UA	--	--	--	--	89.4	233	358	332	10,2	358	510	459	99.6
βCD	13.8	64	115	91	65.7	294	351	331	17.0	390	557	506	99.9
PM	10.3	41	88	75	77.7	209	360	322	5.6	360	554	507	99.8
UA/βCD	5.2	≈28	84	37	78.8	214	354	321	12.5	354	444	611	93.5

a Temperatures are given in °C. Loses of mass lower than 3% have not been recorded. Δ*m*_total_ corresponds to the difference between initial (at environment temperature close to 27 °C) and final mass (at 800 °C) concerning overall range of temperature (including undefined losses of mass lower than 3%).

### 3.4 NMR Analysis

The NMR spectra were valuable tools for identifying inclusion complexes, as they demonstrated the chemical shift changes in the internal protons (H3 and H5) of βCD when a molecule is included in its cavity.^36^Figure S1 depicts the ^1^H NMR spectra of βCD, as described in literature.^37^Figure S2 displays the ^1^H NMR spectra of UA/βCD IC, revealing signals referring to the hydrogens 1 to 6 of both βCD and UA. The signals corresponding to UA were attributed based on literature descriptions.^38^

The data presented in Table S1 reveal that upon the insertion of UA into the βCD cavity, the signals corresponding to βCD in the spectrum of the UA/βCD IC (Figure S6) undergo a change in chemical shift (Δδ H1: 0.0041 ppm; Δδ H2: 0.0020 ppm; Δδ H3: 0.0019 ppm; Δδ H4: 0.0020 ppm; Δδ H5: 0.0016 ppm; Δδ H6: 0.0026 ppm). A significant chemical shift in the signal of H1 in the UA/βCD inclusion complex spectrum (Δδ: 0.0041 ppm) provides evidence of the participation of UA in the inclusion complex. H1 is located on the anomeric carbon (C1) of the glucose units in the βCD structure. This hydrogen is particularly important because it is sensitive to interactions with guest molecules within the βCD cavity.^39^ The observed shift (Δδ: 0.0041 ppm) in the H1 signal is indicative of changes in the electronic environment due to the formation of the inclusion complex.

Changes in chemical shifts upon the formation of inclusion compounds with CD are often related to disturbances in the electronic density of the atoms. These changes are caused by the unbound electrons of the C-1–O-5–C-4 oxygen atoms from the glucosidic bonds of the CD molecules, confirming the occurrence of interactions.^40,41^

Furthermore, the ^1^H NMR spectrum of the UA/βCD PM (Figure S3) shows a less intense chemical shift in the hydrogen atoms H3 and H5, as indicated in Table S2. A comparison of Tables S1 and S2 highlights the greater chemical shift of H3 and H5 in the spectrum of the inclusion complex.

### 3.5 Evaluation of Hydrophobic Nanoprecipitates by DLS and ZP Titrations

Colloidal characterization by DLS and ZP was performed to assess the ability of βCD to modulate the self-aggregation of UA. Figure 3C shows the average hydrodynamic diameter (*D*_h_) measured by DLS. In the figure, the presence of nanometric structures of 160–260 nm can be observed, resulting from the formation of hydrophobic nanoprecipitates (HNPs). Figure 3D shows the ZP titration of UA and UA/βCD in DMSO/water solutions. The HNPs have negative ZP values, which could be attributed to the partial ionization of UA and βCD.

The UA/βCD IC maintained a more stable ZP with increasing titrant concentration, whereas free UA showed a sharp increase in ZP at concentrations between 0.0010 and 0.0015 mM, reaching zero. ZP values close to zero reduce the colloidal stability of the compounds and favor the formation of agglomerates and phase separation. The higher and stable ZP values for the UA/βCD system suggest that βCD increases the colloidal stability of the nanoprecipitate.

Ferreira et al. (2023) also showed that inclusion complexes of βCD and the methanolic extract of *Mitracarpus frigidus* (MFM) increased the colloidal stability of the system compared to MFM alone. These results support the hypothesis that βCD improves the cohesion of the assemblies between the compounds included in its nanostructure.^42^

### 3.6 Solubility Evaluation

**Figure 4:**
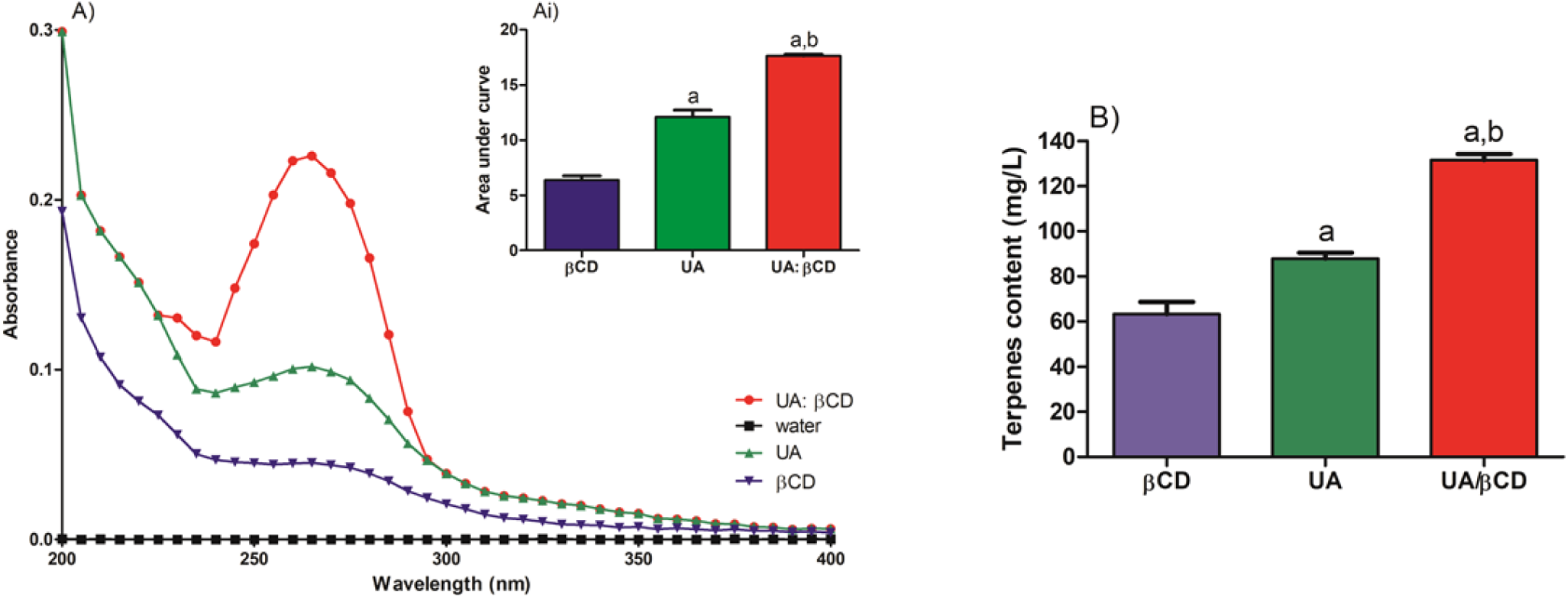
Diagram of the solubility of UA, βCD, and UA/βCD in an aqueous solution. A) UV–vis spectral. Ai) Area under curve of UV–vis spectral. B) Determination of total terpenes. UA, ursolic acid; UA/βCD, UA/βCD inclusion complex; βCD, β-cyclodextrin.

UA is a pentacyclic triterpenoid compound with very low aqueous solubility, approximately 0.102 μg/L.^43^ The interaction between UA and βCD results in stable inclusion complexes, which may increase the aqueous solubility of UA,^44,45^ as shown in Figure 4A.

**Figure 5:**
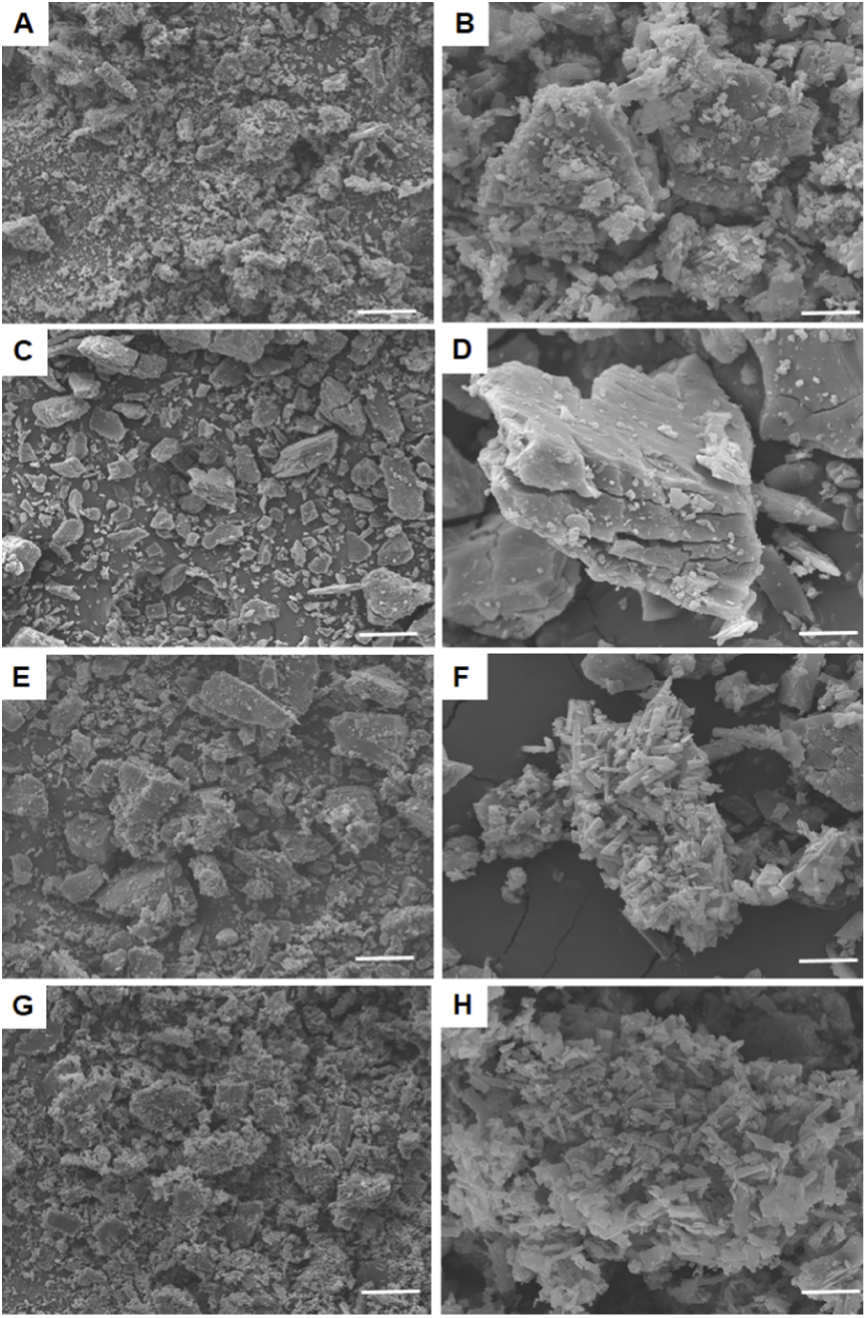
SEM micrographs of UA (A, 500×; B, 3000×), βCD (C, 500×; D, 3000×), PM (E, 500×; F, 3000×), and UA/βCD (G, 500×; H, 3000×). UA, ursolic acid; UA/βCD, UA/βCD inclusion complex; PM, physical mixture between βCD and UA; βCD, β-cyclodextrin.

The solubilization tests showed that the IC had a statistically higher solubility compared to UA and βCD, as indicated by the area under curve (AUC) values (UA: 12.72; βCD: 6.78; UA/βCD: 17.28). (Figure 5Ai, *p* < 0.05). These results suggest that the IC increased the solubility of UA by approximately 35.85%. The terpenoid content dosage supports this hypothesis, as the terpenoid content of the UA/βCD aqueous solution after filtration is higher (131.58 ± 5.97 mg/L) compared with the terpenoid content of the UA solution (87.91 ± 5.80 mg/L) (Figure 4B).

These findings are supported by Lateh et al. (2022) who also observed an increase in water solubility up to 55.6 ± 2.6 μg/mL of a curcuminoid-rich extract when complexed with hydroxypropyl-β-cyclodextrin and polyvinylpyrrolidone K30.^44^ Thus, these results show that βCD complexation increases the solubility of UA in an aqueous solution.

### 3.7 SEM Analysis

Figure 5 shows the micrographs of UA, βCD, UA/βCD, and PM. UA and βCD appeared as irregularly shaped crystals (Figure 5A–D). The physical mixtures resulted in a morphology similar to that of βCD and UA separately. This phenomenon showed that both were just mixed together in a heterogeneous matrix. On the other hand, a drastic change in the morphology of UA-βCD was observed compared to βCD and UA (Figure 5G,H). Such changes in particle shape, size, and appearance, as well as the formation of agglomerates, are due to the different colloidal characteristics of IC and free UA, which should have a different crystallization profile due to different interactions, resulting in microstructural differences in the solid state.^46^

### 3.8 Biological Studies

This study was designed as a preliminary screening; we limited the assays to a single concentration (30 μg/mL) to confirm the biological activity of the inclusion complex and assess its potential for further development. It is noteworthy that determining the IC50 value is essential for deeper characterization and must be considered to optimize the formulation and evaluate its detailed dose-response behavior. The concentration of 30 μg/mL and the exposure time of 24 h were chosen based on preliminary screening experiments. These parameters were selected to ensure that the biological activity of UA and UA/β-CD complexes could be evaluated at a concentration likely to exhibit anticancer effects, based on literature reports for similar compounds. For instance, Kang et al. (2012)^47^ evaluated the cytotoxic effects of UA in human cancer cell lines at concentrations between 20 and 40 μg/mL, with an exposure time of 24 h, observing significant inhibition of cell proliferation. Similarly, Wozniak et al. (2015)^48^ conducted cytotoxicity studies on different cancer cell lines with UA. Their results showed significant anticancer effects at concentrations ranging from 10 to 50 μg/mL, supporting the use of similar concentrations for preliminary screening.^48^

More recently, de Souza et al. (2020)^49^ employed similar concentrations of 25–50 μg/mL in their study on the inclusion complex of UA with β-CD to assess its solubility enhancement and anticancer effects on different cell lines. Similarly, Shao et al. (2019)^50^ tested UA-loaded nanoparticles at concentrations between 10 and 30 μg/mL and demonstrated effective cytotoxicity against breast cancer cells after 24 h of treatment, aligning with the conditions used in this work.

**Figure 6:**
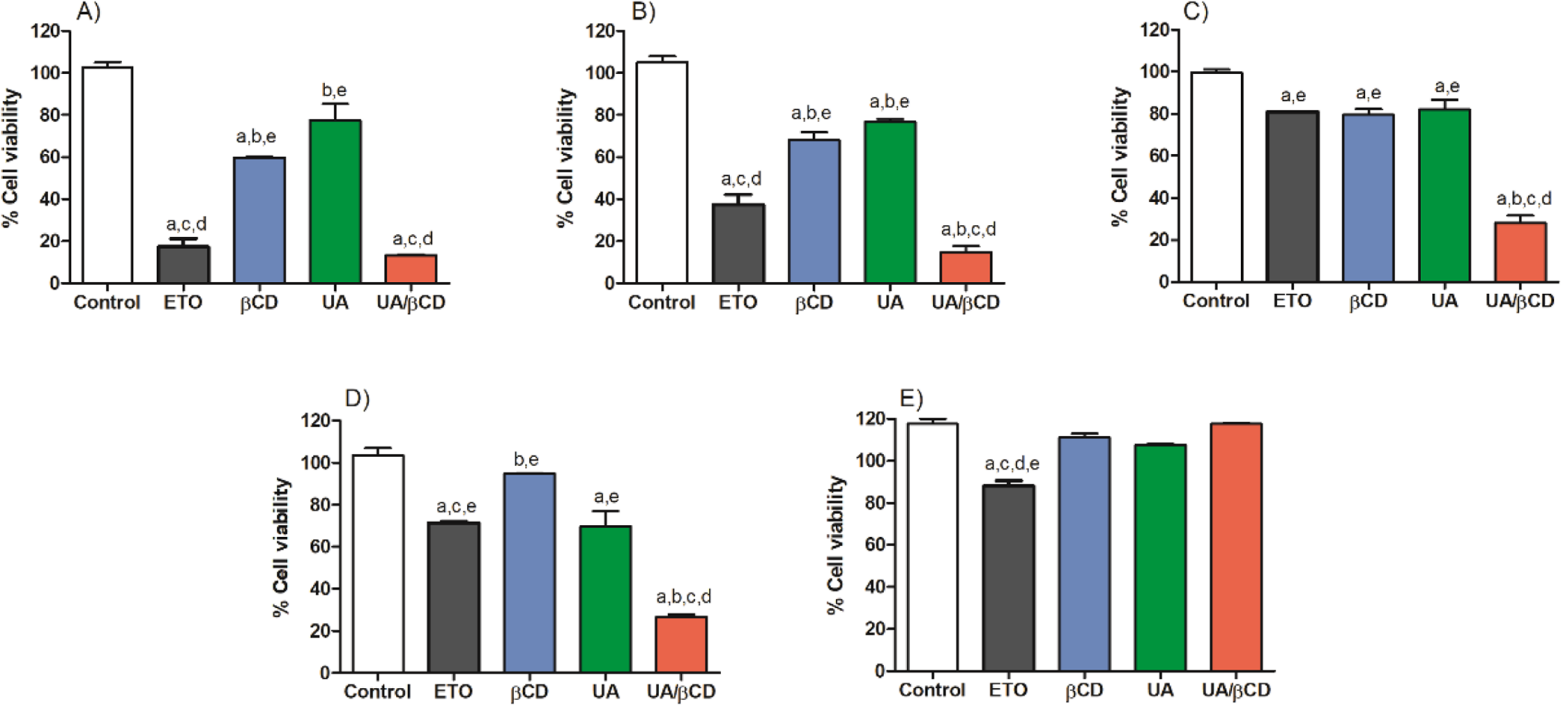
Evaluation of *in vitro* antitumor activity in cancer and normal cell lines treated with UA, βCD, UA/βCD, and ETO by the MTT assay. A) HL60; B) JURKAT; C) MDA; D) MCF-7; and E) VERO. Results were expressed as mean ± standard deviation of three independent experiments. a, statistical difference from control; b, statistical difference from etoposide; c, statistical difference from βCD; d, statistical difference from ursolic acid; e, statistical difference from UA/βCD complex. UA/βCD, UA/βCD inclusion complex; UA, ursolic acid; βCD, β-cyclodextrin; ETO, etoposide.

Generally, for cytotoxic MTT assays, treated cancer cell lines are expected to show cell viability below 70%. The results (Figure 6) revealed that UA/βCD reduced the cell viability of both HL60 and JURKAT tumor cell lines, similarly to etoposide (86.9 ± 0.84% and 85.35 ± 4.03%). However, better antitumor activity was observed for both MDA and MCF-7 cell lines compared to etoposide (71.95 ± 4.88% and 73.40 ± 1.55%). It was also observed that UA/βCD exhibited more pronounced antitumor activity compared to free UA.

Complexation with CD increased the antitumor activity of UA, probably by improving UA solubility. These findings are supported by Shukla et al. (2020), who showed that the complexation of cyclodextrin with celastrol, a pentacyclic triterpenoid, resulted in increased cytotoxicity in human lung cancer cells and improved aqueous solubility compared to free celastrol (*p* < 0.0001).^51^

It can also be observed that UA and UA/βCD showed no cytotoxicity to VERO cells (normal cell line), being considered less toxic than etoposide. βCD showed a reduction in cell viability only for HL60 and MDA. As for other cell lines, cell viability remained above 70%. The activity of UA/βCD can be explained by the activity of UA, which is considered a potent antitumor agent. Reports found in the literature show that this triterpene induces apoptosis and prevents the proliferation of cancer cells.^52^

The MIC assay was also performed as part of a preliminary screening to confirm the antibacterial activity of free UA and UA/βCD IC. For this purpose, three Gram-positive bacteria (*E. faecalis*, *S. aureus*, and *B. cereus*) and two Gram-negative bacteria (*K. pneumoniae* and *E. coli*) were selected. The UA/βCD IC showed a significant MIC value against all tested bacterial species, except for *E. coli* (Table 2). It was observed that the incorporation of UA with βCD resulted in a decrease in MIC values and consequently improved antibacterial activity, mainly against *E. faecalis* (UA MIC: 31.3 μg/mL; UA/βCD MIC: 7.8 μg/mL), followed by *S. aureus*, *B. cereus*, and *K. pneumoniae* (UA MIC: 31.3 μg/mL; UA/βCD MIC: 15.6 μg/mL). Marques et al. (2019)^53^ also showed that CD inclusion complexes with the essential oil of *Pimenta dioica* were able to increase the antimicrobial activity against *S. aureus*, *E. coli*, *Listeria monocytogenes*, *Pseudomonas aeruginosa*, and *Salmonella enteritidis* compared to the essential oil alone (*p* < 0.05).^53^

**Table 2:** *InVitro* Antibacterial Activity of UA, UA/βCD, βCD, and Positive Controls against Bacterial Species^a^

MIC values (μg/mL)					
	*S. aureus*	*B. cereus*	*K. pneumoniae*	*E. faecalis*	*E. coli*
	ATCC 6538	ATCC 14579	ATCC 4552	ATCC 19433	ATCC 10536
UA	31.3	31.3	31.3	31.3	>1000
UA/βCD	15.6	15.6	15.6	7.8	>1000
βCD	>1000	>1000	>1000	>1000	>1000
Ciprofloxacin	0.39	0.20	12.5	1.56	3.13
Chloramphenicol	12.5	3.13	12.5	6.25	0.20

a UA: ursolic Acid; UA/βCD: UA and βCD inclusion complex; βCD: β-cyclodextrin.

These results may be related to the increased solubility and bioavailability of the compounds after encapsulation. It is noteworthy that the MIC values presented by UA/βCD produced a bacteriostatic effect, which is corroborated by previous studies reported in the literature.^54^

## 4 Conclusion

This study reported a multifaceted approach to investigate the interaction between UA and βCD with a focus on spectroscopic analyses, thermal studies, and colloidal characterization. The purpose of this study was to assess the potential of the IC as an anticancer agent as a first step toward further development. UA is a pentacyclic triterpenoid that exhibits poor aqueous solubility. Thus, to overcome this limitation, the IC with β-CD was prepared, which has been shown to significantly improve UA’s solubility by 35.85%. The enhanced solubility of UA in the IC was verified through solubility tests, confirming its potential for increased bioavailability.

Molecular docking simulations provided insightful predictions of a favorable interaction between UA and the internal cavity of βCD. Subsequent experimental validations by FTIR and ^1^H NMR spectroscopies suggest specific interactions involving the carbonyl group of UA and the cavity of βCD. Thermal analyses and colloidal studies supported the successful formation of the IC. Additionally, our results demonstrated promising selective anti-tumor activity of IC against different cancer cell lines and enhanced antimicrobial effects, suggesting potential applications in drug delivery systems.

At this stage, the focus was on characterizing and determining whether the complex displayed sufficient activity to justify further investigation. The determination of IC50 values will be a key part of our future research in the development of the final formulation of the inclusion complex. This next step involves more detailed dose-response studies across multiple concentrations, enabling us to establish precise IC50 values for each cell line. Future research will also explore the phase solubility, release mechanisms, and kinetics of the inclusion complex to optimize its formulation for therapeutic applications. Our current results provide a foundation for this future work, which aligns with the scope of the present study. In summary, our research sheds light on the potential of βCD as a promising carrier for UA in pharmaceutical formulations, offering opportunities for the development of innovative drug delivery systems to improve the solubility, dissolution rate, and bioavailability of poorly water-soluble drugs like UA.

## Data Availability

All the data from this work are available in the paper. The authorization for the use of the plant species is registered in SISGEN/Brazil - A032F41.

## Supporting Information Available

^1^H NMR spectra βCD; ^1^H NMR spectra βCD/UA inclusion complex; ^1^H NMR spectra βCD/UA physical mixture; assignment and chemical shift of the 1H of free βCD, UA/βCD physical mixture, and UA/βCD inclusion complex in DMSO-*d*_6_ at 25 °C and variation in chemical shift values of hydrogens of the βCD molecule (PDF)

## Abbreviation

CD: cyclodextrin
*D*_h_: hydrodynamic diameter
DLS: dynamic light scattering
DMSO: dimethyl sulfoxide
DTA: differential thermal analysis
ETO: etoposide
FBS: fetal bovine serum
HNPs: hydrophobic nanoprecipitates
IC: inclusion complex
MBC: minimum bactericidal concentration
MIC: minimal inhibitory concentration
PM: physical mixture
MTT: 3-(4,5-dimethyl-2-thiazolyl)-2,5-diphenyl-2*H*-tetrazolium bromide
SEM: scanning electron microscope
TGA: thermogravimetric analysis
UA: ursolic acid
ZP: zeta potential
