# Supplementary material for: Enhanced Antitumor and Antibacterial Activities of Ursolic Acid through β-Cyclodextrin Inclusion Complexation
Source: ACS Omega. 2025 Mar 26;10(13):12906–16. doi: 10.1021/acsomega.4c08337 (PMC11983339; doi:10.1021/acsomega.4c08337)
Supplement: Supplementary file 1 — ao4c08337_si_001.pdf [file ao4c08337_si_001.pdf]

Enhanced Antitumor and Antibacterial Activities of Ursolic Acid Through β-Cyclodextrin Inclusion Complexation

Júlia B. Fajardo[a], Mariana H. Vianna[a], Thayná G. Ferreira[a], Ari S. de O. Lemos[a], Thalita de F. Souza[a], Lara M. Campos[a], Priscila de L. Paula[a], Nubia B. Andrade[a], Lívia R. Gamarano[a], Lucas S. Queiroz[b], Bruno de A. Oliveira[c], Adilson D. da Silva[c], Luciana M. Chedier[d], Ângelo M. L. Denadai[e], Guilherme D. Tavares[f], Thaís N. Barradas[f], Rodrigo L. Fabri[a*]

[a] Laboratory of Bioactive Natural Products, Department of Biochemistry, Institute of Biological Sciences, Federal University of Juiz de Fora, Juiz de Fora, Minas Gerais, Brazil.

[b] Research Group for Food Production Engineering, National Food Institute, Technical University of Denmark, Ørsteds Plads, Kongens Lyngby, Denmark.

[c] Department of Chemistry, Institute of Exact Sciences, Federal University of Juiz de Fora, Juiz de Fora, Minas Gerais, Brazil.

[d] Department of Botany, Institute of Biological Sciences, Federal University of Juiz de Fora, Juiz de Fora, Minas Gerais, Brazil.

[e] Department of Pharmacy, Institute of Life Sciences, Federal University of Juiz de Fora, Campus Governador Valadares, Governador Valadares, Minas Gerais, Brazil.

[f] Department of Pharmacy, Faculty of Pharmacy, Federal University of Juiz de Fora, Juiz de Fora, Minas Gerais, Brazil.

*Corresponding author. Bioactive Natural Products Laboratory, Department of Biochemistry, Biological Sciences Institute, Federal University of Juiz de Fora, Juiz de Fora, Minas Gerais CEP 36036-900, Brazil. E-mail address: rodrigo.fabri@ufjf.br (R.L. Fabri).

Supplementary material

Figure S1 – ^{1}H NMR spectra βCD. βCD : ^{1}H NMR (500 MHz, DMSO-d_6) δ 5.74 (d, J = 6.9 Hz, H1), 5.68 (s, H1), 4.82 (d, J = 3.6 Hz, H1, 4.47 (s, H1), 3.63 (t, H2), 3.59 (s, H1), 3.56 (d, J = 9.7 Hz, H1), 3.31 (bs, H1).

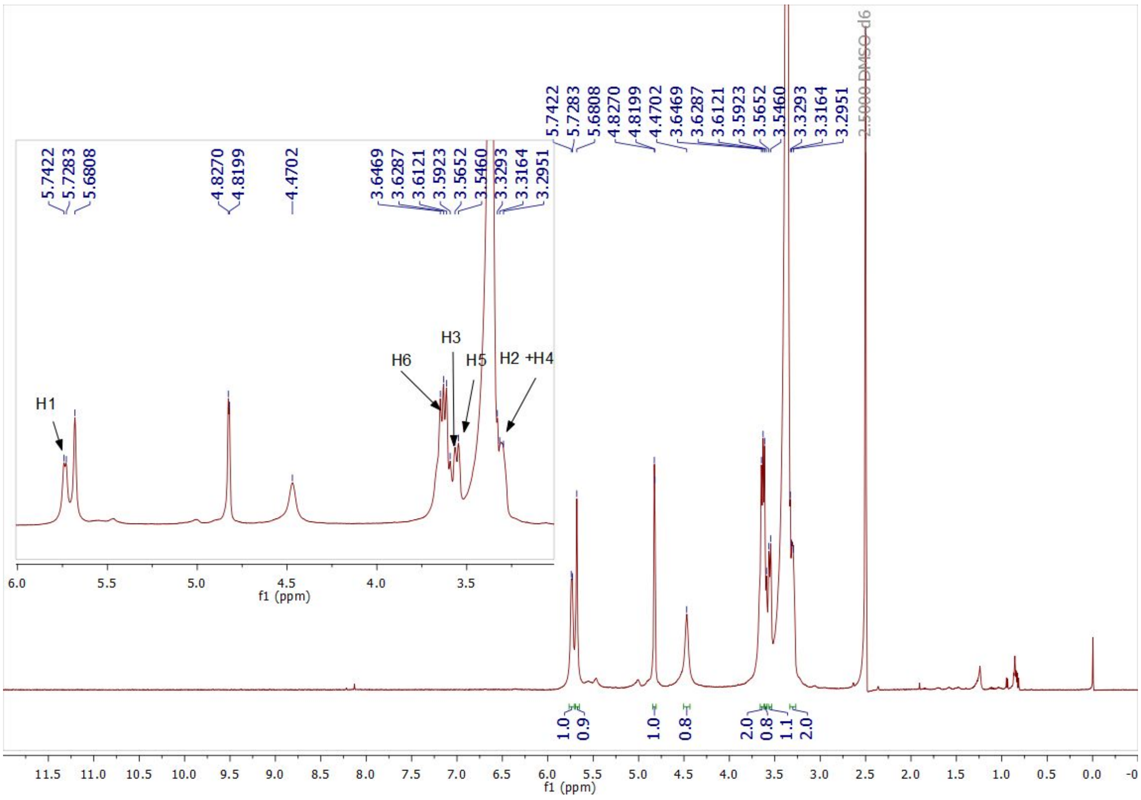

Figure S2 – ^{1}H NMR spectra βCD/UA inclusion complex. UA/βCD inclusion complex: 1H NMR (500 MHz, DMSO-d6) δ 5.74 (d, J = 6.9 Hz, H14), 5.69 (s, H14), 5.12 (s, H1), 4.82 (d, J = 3.5 Hz, H14), 4.46 (t, J = 5.7 Hz, H14), 4.30 (d, J = 5.1 Hz, H1), 3.63 (t, H28), 3.59 (d, J = 5.6 Hz, H7), 3.56 (d, J = 9.3 Hz, H14), 3.30 (bs, H14), 3.00 (dd, J = 10.3, 5.9 Hz, H1), 2.10 (d, J = 11.4 Hz, H1), 1.97 – 1.76 (m, H4), 1.50 (m, J = 35.4, 10.7 Hz, H10), 1.35 – 1.18 (m, H4), 1.04 (s, H3), 0.93 – 0.84 (m, H15), 0.81 (d, J = 6.5 Hz, H3), 0.75 (s, H3), 0.67 (s, H3).

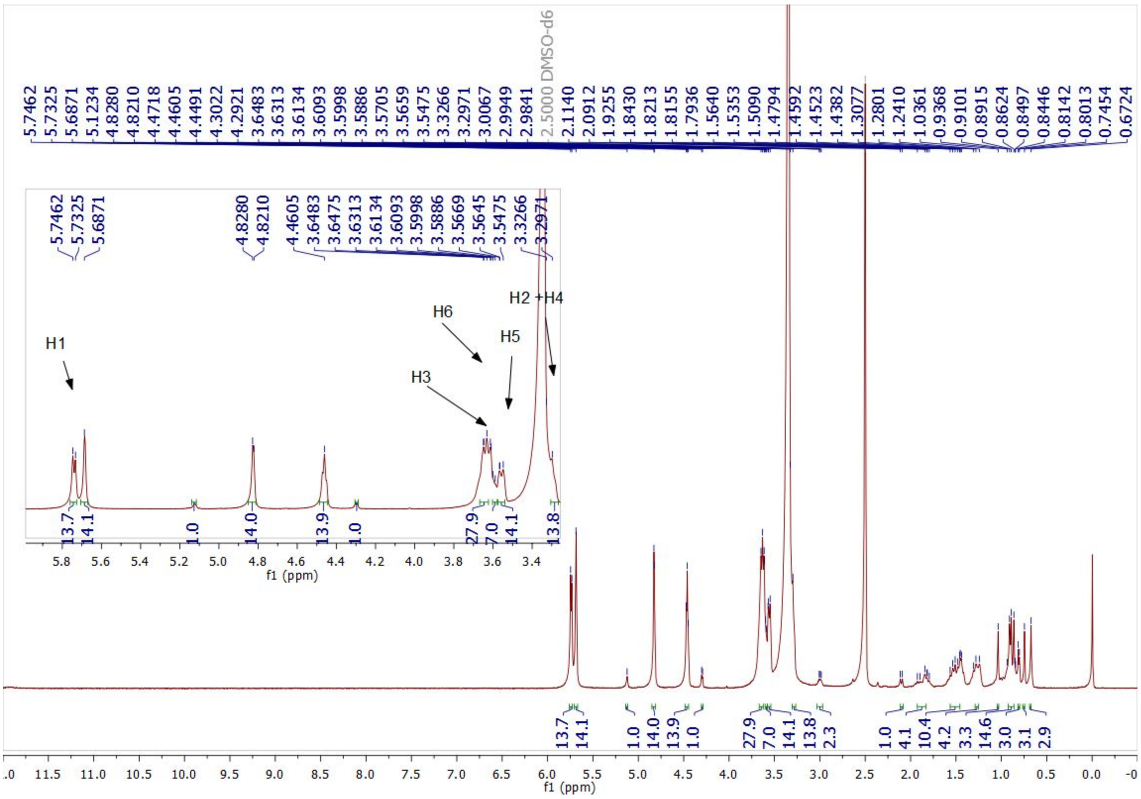

Figure S3 - ^{1}H NMR spectra βCD/UA physical mixture. UA/βCD PM: ^{1}H NMR (500 MHz, DMSO-d_6) δ 5.74 (d), 5.68 (s), 5.12 (s), 4.82 (d, J = 3.6 Hz), 4.47 (s), 3.62 (t), 3.59 (s), 3.56 (d, J = 9.6 Hz), 3.30 (bs), 3.00 (m), 2.10 (d, J = 11.2 Hz), 1.98 – 1.72 (m), 1.50 (dd, J = 38.7, 21.9, 8.7 Hz), 1.35 – 1.18 (m), 1.04 (s), 0.96 – 0.82 (m), 0.81 (d, J = 6.6 Hz), 0.74 (s), 0.67 (s).

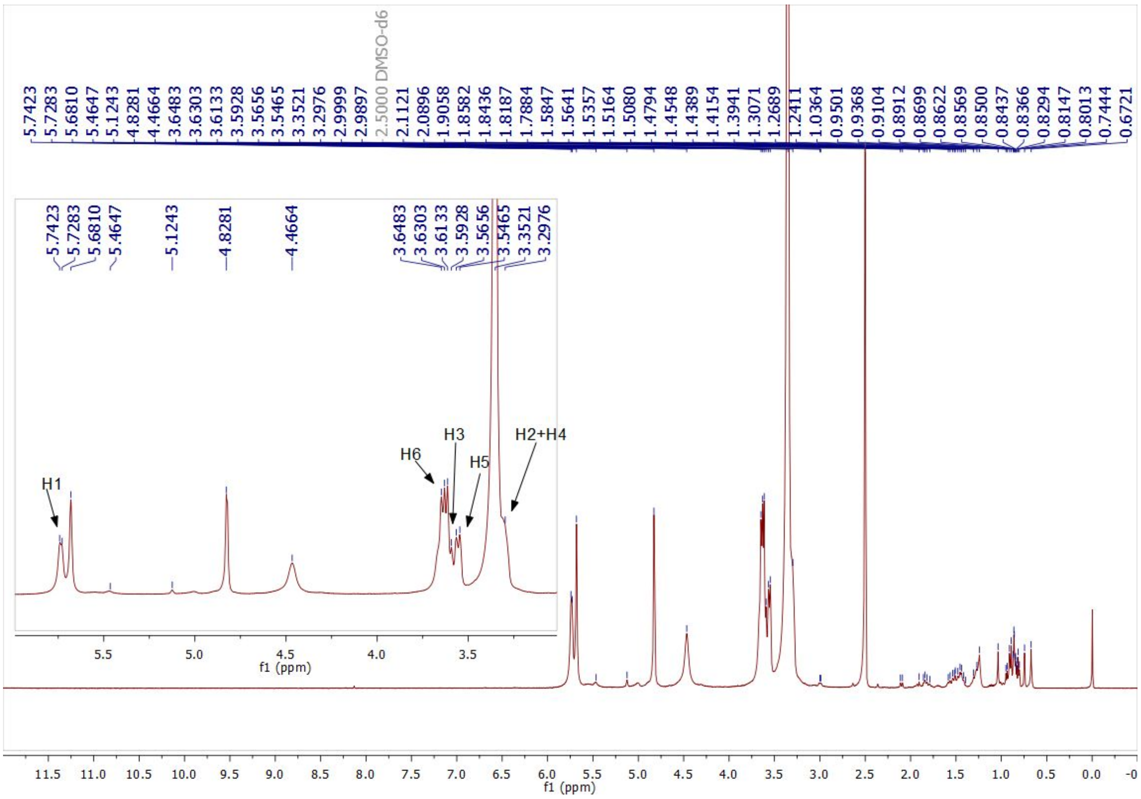

Table S1- Assignment and chemical shift of the 1H of free βCD and UA/βCD inclusion complex in DMSO-d6 at 25 °C and variation in chemical shift values of hydrogens of the βCD molecule.

	βCD(ppm)	UA/βCD(ppm)	Δδ(ppm)
H1	5.7353	5.7394	0.0041
H2	3.2951	3.2971	0.0020
H3	3.5923	3.5942	0.0019
H4	3.2951	3.2971	0.0020
H5	3.5556	3.5572	0.0016
H6	3.6287	3.6313	0.0026

Table S2- Assignment and chemical shift of the ^{1}H of βCD and UA/βCD physical mixture in DMSO-d6 at 25 °C and variation in chemical shift values of hydrogens of the βCD molecule.

	βCD(ppm)	PM (ppm)	Δδ(ppm)
H1	5.7353	5.7353	0.0000
H2	3.2951	3.2976	0.0025
H3	3.5923	3.5928	0.0005
H4	3.2951	3.2976	0.0025
H5	3.5556	3.5560	0.0004
H6	3.6287	3.6303	0.0016
